# The effect of rosemary Extract and cold plasma treatments on bacterial community diversity in poultry ground meats

**DOI:** 10.1016/j.heliyon.2019.e02719

**Published:** 2019-10-25

**Authors:** Hung-Yueh Yeh, John E. Line, Arthur Hinton, Yue Gao, Hong Zhuang

**Affiliations:** a Poultry Microbiological Safety and Processing Research Unit, U.S. National Poultry Research Center, Agricultural Research Service, U.S. Department of Agriculture, 950 College Station Road, Athens, GA 30605-2720, USA; b Quality and Safety Assessment Research Unit, U.S. National Poultry Research Center, Agricultural Research Service, U.S. Department of Agriculture, 950 College Station Road, Athens, GA 30605-2720, USA; c National Center of Meat Quality and Safety Control, Nanjing Agricultural University, Nanjing, 210095, China; d Suzhou Polytechnic Institute of Agriculture, Suzhou, 215008, China

**Keywords:** Microbiology, Food microbiology, Biodiversity, Bacteria, Microorganism, Metabolite, Bacterial community, Cold plasma, Single carbon sources, EcoPlates, Poultry ground meat

## Abstract

To provide safer food, many technologies have been used to preserve food. One such technology is cold plasma, which can reduce viable bacterial counts in various food matrices. However, bacterial communities in food matrices before and after cold plasma treatment have not been investigated. In this communication, the EcoPlates™ were used to physiologically profile bacterial communities from poultry ground meat treated with rosemary, cold plasma or both. The cultures in the plates were incubated at 25 °C for seven days in an OmniLog® system. Responses of the bacterial communities to 31 chemicals were measured on formazan production. The results show that the three parameters of the Gompertz growth curves were observed in all samples, 2-hydroxybenzoic acid could not be used, while pyruvic acid methyl ester was used for a carbon source by the bacterial communities from all meat samples, each bacterial community metabolized different numbers of chemical compounds at different rates, and reduction of bacterial functional diversity was observed in the poultry meat samples treated with cold plasma and rosemary. In the future, investigations on whether the physiological profiling in bacterial communities be used as an indicator for effectiveness of cold plasma treatment of meat samples.

## 1 Introduction

Food safety is one of important health issues worldwide. In order to prevent food spoilage and preserve food quality and sanitary conditions for human consumption, many physical, chemical and biological treatments of food with various technologies have been explored [1, 2, 3]. One such technology is plasma, the fourth fundamental state of matter, that consists of highly charged particles and unbound electrons [4, 5]. Based on the temperature of the medium, plasma can be classified into cold and hot plasmas [4, 6, 7]. The latter means the temperature of the electrons, the ions and the neutrals are all the same, and the gas molecules in the hot plasma are fully ionized [4, 6]. The former indicates the temperature of the ions and neutrals is lower than that of the electrons [4, 6]. In addition, only a small fraction of the gas molecules is ionized [4, 6].

Due to its non-thermal, versatile and economical natures [8], cold plasma technology has been widely used by food industries ([8, 9] for reviews). One such usage is to inactivate foodborne pathogens in various food matrices [7, 10, 11, 12]. These results demonstrate that this technology is effectively able to reduce viable foodborne pathogens as well as spoilage microorganisms [7, 12, 13, 14, 15, 16, 17, 18]. Consequently, these results may be very useful for improving food safety and extending food shelf life. Rosemary is a good source of natural antioxidant so that it has been added in food processing to extend the food shelf-life [19, 20, 21]. However, microbial compositions or communities in food matrices have not been extensively explored. Analysis of bacterial communities in poultry ground meats allows us to predict the effectiveness of antimicrobial or other treatments to control microbiological safety and quality. One such the most common approach is community-level physiological profiling [22, 23, 24] using EcoPlates™ (Biolog Inc., Hayward, CA, USA).

The aim of this study was to investigate the bacterial communities in poultry ground meat after treatment with rosemary extract, cold plasma and combination of both. The EcoPlates™ were used to assess the bacterial catabolic activity and diversity in meat samples after treatment, and to determine the relationships of the patterns of metabolic diversity and efficiency of the treatment.

## 2 Materials and methods

### 2.1 Poultry meat samples

Raw boneless skinless chicken breast fillets (*Pectoralis major*) were purchased from a local grocery store, and transported on ice in a cooler to the laboratory in the U.S. National Poultry Research Center, Athens, GA, USA. In the laboratory, the meat samples were stored at 4 °C for 24 h before they were ground (MEGAFORCE® 3000 Series, STX International, Lincoln, NE, USA) for patties as described previously [12, 18]. For rosemary treatment, 495 g of ground meat was mixed with 5 g (1%) rosemary (*Rosmarinus officinalis*) extract for 5 min at a speed setting of “2” using a kitchen mixer (K45SS, KitchenAid Inc., Benton Harbor, MI, USA). Poultry meat samples without treatment of rosemary extract were prepared as controls. The ground meat samples were made into patties of 15 g each with 5 mm depth x 50 mm diameter using a template. Each patty was then placed into a 15 mm depth x 60 mm diameter dish (Corning Life Sciences, Tewksbury, MA, USA).

### 2.2 Sample packaging

Two individual dishes (each with a 15 g patty) were placed in a Cryovac CS977 polymer tray (Sealed Air Corp., Duncan, SC, USA). The trays were filled with modified atmosphere (MA) gas (65% O_2_, 30% CO_2_, and 5% N_2_, respectively) using a gas mixer (Gas Mixer KM-Flow, Witt-Gasetechnik GmbH and Co KG, Witten, Germany), and sealed with a polypropylene film (Toplex HB60, Plastopil Inc., Maywood, NJ, USA) by a tray sealer (KOCH Kats 100 Single Head Tray Sealer, UltraSource LLC., Kansas City, MO, USA). The gas compositions of the packages were verified with a headspace gas analyzer (CheckPoint-Handheld Gas Analyzer, Ringsted, Denmark), and the actual headspace compositions were 63.2 ± 2.4% O_2_ and 30.6 ± 2.2% CO_2_, respectively. After sealing, the samples were placed at 4 °C for 1 h to let relative humidity in the packages reach about 80% before cold plasma treatment.

### 2.3 In-package dielectric barrier discharge-cold plasma (DBD-CP) treatment

The DBD-CP system for in-package treatment was described previously [7, 12, 17, 18]. In brief, the packaged tray with meat samples in center was placed directly between electrodes and treated with DBD at 70 kV for 180 s. Treatments were performed at ambient temperature of 22 ± 1 °C and relative humidity of 58 ± 1%. Ozone formation in packages was measured immediately after treatment using a spectral method and used as an indicator of DBD treatment consistency, effectiveness, and antimicrobial activity in packages.

### 2.4 Preparation of meat samples for EcoPlates™

Ground poultry meat from above treatment was prepared by addition of sterile phosphate buffered saline (PBS) to make a 10% (v/v) suspension, which was mixed thoroughly by vortex at the maximum speed for 3 min (Fisher Vortex Genie 2, Fisher Scientific, Bohemia, NY, US) at 25 °C. After 10 min on ice, the supernatants were further diluted with PBS to have a final 1% (v/v) concentration [25, 26] that were then dispensed in 150 μl volumes per well into 96-well EcoPlates™ (Biolog Inc.). The plates with lids were incubated in an OmniLog System (Biolog Inc., Hayward, CA, USA) at 25 °C for 168 h. This typical 96-well microplate consists of 31 unique chemicals and a water control in triplicates. The tetrazolium dye in each well was converted into insoluble violet formazan after bacterial respiration. The concentration of the formazan was proportional to the degree of respiration by the microorganisms in the communities.

### 2.5 Data collection

The rate of utilization of the chemicals in the EcoPlates™ as a single carbon source was measured by the production of violet formazan at a wavelength of 590 nm. The optical density (OD) readings were recorded every 30 min by a plate reader associated with the OmniLog System, and the data were exported as an Excel file for statistical analysis. The OD readings of the chemicals were adjusted by the subtraction from the average of the OD values of the water blank in the plates. Mean of the resulting OD values for each chemical compound triplicates was calculated for further analysis. Negative OD values were set to zero after blanking for subsequent data analysis [22, 27]. Richness (*S*), Shannon diversity (*H*) and Shannon evenness (*E*) indices of bacterial functional diversity among the meat samples were calculated as previously described [23, 24].

### 2.6 Statistical analysis

The PAST software package (version 3.22, [28]) was used to determine the bacterial growth curves based on Gompertz growth algorithm, and principal coordinates analysis based on the Euclidean similarity index. GraphPad Prism® 7 (GraphPad Software, La Jolla, CA, USA) was used for data statistical analysis.

## 3 Results and discussion

Ground meat samples were divided into treatment groups as follows (triplicate per group): (1) meat with no treatment as a control (C), (2) meat with cold plasma treatment (CP), (3) meat with rosemary treatment (R) and (4) meat with rosemary and cold plasma treatment (RP). For ozone formation in the samples, there were no differences in ozone concentrations between rosemary-treated samples and control (without rosemary treatment) samples (271 ± 20 ppm and 264 ± 21 ppm, respectively). Treatments of poultry meat samples for EcoPlates™ analysis in this study are listed in Table 1.

**Table 1:** Growth kinetics and indices of metabolic diversity of bacterial communities of ground poultry meat samples from EcoPlates™ assay.

Groups	Rosemary^1^	Cold Plasma^1^	Storage (Day) 1	Growth Rate (Hour)^2^	Maximum Population Size^2^	*H*^3^	*E*^3^	*S*^3^
C0	-	-	0	0.03674 ± 0.002949^4^	72.68 ± 17.72	3.016 ± 0.1017	1.285 ± 0.054	10.560 ± 0.801
R0	+	-	0	0.04175 ± 0.005297	54.70 ± 11.08	2.857 ± 0.1062	1.103 ± 0.083	13.780 ± 1.788
CP0	-	+	0	0.03474 ± 0.002937	78.31 ± 14.68	2.926 ± 0.1096	1.279 ± 0.077	10.560 ± 2.058
RP0	+	+	0	0.03258 ± 0.003411	54.65 ± 9.63	2.844 ± 0.0773	1.290 ± 0.048	9.333 ± 1.202
C5	-	-	5	0.0502 ± 0.001484	162.40 ± 1.15	3.310 ± 0.0174	1.024 ± 0.013	25.440 ± 1.495
R5	+	-	5	0.03806 ± 0.004567	117.50 ± 9.27	3.251 ± 0.0589	1.010 ± 0.010	25.220 ± 1.966
CP5	-	+	5	0.0331 ± 0.002696	79.54 ± 17.49	2.966 ± 0.0868	1.184 ± 0.019	12.440 ± 1.352
RP5	+	+	5	0.03658 ± 0.001038	71.21 ± 5.62	2.888 ± 0.0485	1.092 ± 0.039	14.440 ± 1.788

1 Treatments of ground meat samples: -, no treatment; +, treatment as indicated; and 0 and 5, the samples were stored at 4 °C for 0 and five days, respectively.

2 The growth data based on the three-parameter Gompertz growth model were calculated with the PAST software package [28]. The formula is $f\left(t\right)=\;ae^{-be^{-ct}}$. *a*, an asymptote (also called maximum population size); *b*, a displacement of the *x* axis; *c*, the growth rate.

3 *H*: Shannon-Weiner functional diversity index, *E*: Shannon evenness index, and *S*: catabolic richness. They were calculated according to Jałowiecki *et al*. [23] and Grządziel *et al*. [24].

4 Mean ± SEM, (*n* = 3).

**Fig. 1:**
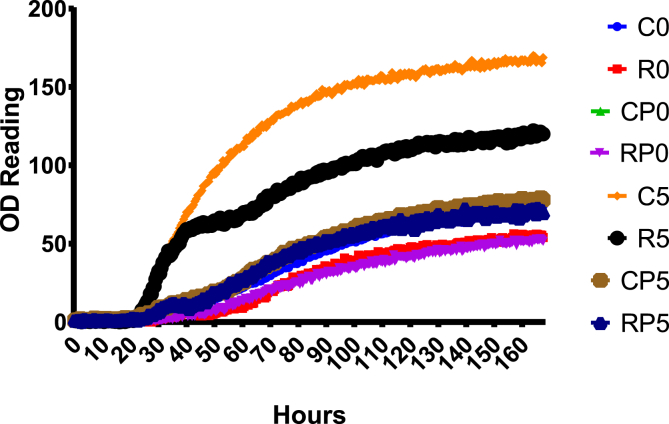
Growth kinetics of bacterial communities from poultry ground meat samples with different treatments. The *x*-axis indicates the culture incubation time in hours, and *y*-axis indicates OD at the wavelength of 590 nm. Samples treatments are indicated at the right.

As depicted in Fig. 1, the growth kinetics of bacterial communities from ground meat samples followed the Gompertz sigmoidal model for the 168-hour incubation period, including a lag phase, an exponential phase and a stationary phase. The growth data fitted the three parameters of the Gompertz growth equation,

$$f\left(t\right)=\;ae^{-be^{-ct}}$$

where *a* is an asymptote (also called maximum population size), *b* is a displacement of the *x* axis, *c* is the growth rate, an *e* is a Euler's number. The highest asymptote was the C5 group (*a* = 162.40, 95% confidence interval [CI95%] ranges, 161.6–163.2), followed by the R5 group (*a* = 117.50, CI95% ranges, 116.5–118.5). The lowest asymptote was the RP0 group (*a* = 54.65, CI95% ranges, 54.03–55.16) (Table 1, Fig. 1). It is also observed that the C5 group (*c* = 0.0502, CI95% ranges, 0.04853–0.05171) had the highest growth rate, while the RP0 group (c = 0.03258, CI95% ranges, 0.0179–0.04726) had the slowest growth rate (Table 1). Similar growth patterns have been reported earlier by others [e.g. 22, 26, 27, 29]. Specifically, the maximum population sizes of the C5 group was statistically significantly higher than the C0 group after five-day storage at 4 °C. This is also true in the R5 vs. R0 groups. However, there were no statistically significant differences in the CP0 vs. CP5 and RP0 vs. RP5 groups. Further analysis on population sizes of bacterial communities of the samples after five-day storage at 4 °C, there were statistically significant differences in the C5 vs. CP5 and C5 vs. RP5 groups, but not the C5 vs. R5 groups. These results of growth kinetics in the bacterial communities from the poultry ground meat samples with various treatments suggest that (1) as expected, bacteria in the meat samples were able to continue to grow during storage at 4 °C, and (2) the cold plasma treatment effectively reduce or inactivate the bacterial population sizes in the meat samples tested after the five-day storage at 4 °C. These results are in an agreement with previous reports on reduction of the numbers of the total viable aerobic counts in poultry meat samples after the cold plasma treatment [7, 12, 18]. However, the result of this study shows rosemary extract did not inactivate very effectively in the meat samples after the five-day storage.

Next, the data obtained from the end-point incubation of each group were analyzed for functional diversity in bacterial communities of the poultry ground meat with various treatments. As depicted in Table 1, the distinctive differences in bacterial metabolic activity were found among poultry ground meat samples treated with different methods. The Shannon-Weiner functional diversity index (*H*) ranged from 2.844 to 3.310, which is in an agreement with ranges between 1.5 and 3.5. The highest value of *H* was from the C5 group, while the lowest value of *H* was from the RP0 group. The chemical richness (*S*) in the meat samples ranged from 9.353 to 25.440 with the highest value from the C5 group and the lowest value from the RP0 group. The third index (*E*, chemical evenness index) ranged from 1.010 to 1.290. The RP0 group had the highest value, while the lowest index value was found in the R5 group. Specifically, there was a statistically significant difference in the C5 vs. RP5 group. Although it was not statistically significant, it is possible that the difference in their bacterial communities between the C5 and CP5 groups could still exist. These results suggest that the reduction of the functional diversity in bacterial communities occurred in samples treated with rosemary and cold plasma.

**Fig. 2:**
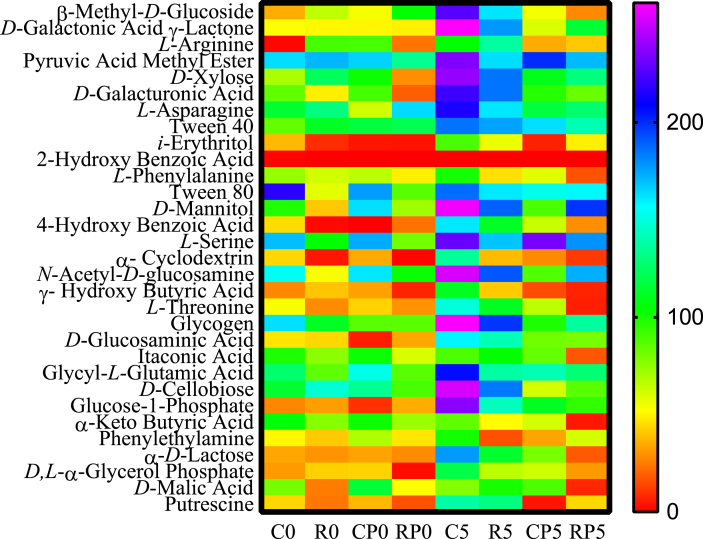
Heatmap diagram of utilization of chemical compounds by bacterial communities in the poultry ground meat samples. The left *y*-axis indicates the chemical compounds used in the EcoPlates™, and the right *y*-axis indicates the scales of OD at the wavelength of 590 nm. The *x*-axis indicates the treatments of poultry ground meat samples as in the Fig. 1 legend.

The use of the chemical compounds as single carbon sources by the bacterial communities from poultry ground meat samples was assessed. As depicted in Fig. 2, the bacterial communities in meat samples could use various chemicals as the carbon sources at various degrees. The bacterial community from the C5 group could use 30 chemical compounds except 2-hydroxybenzoic acid. The bacterial communities from the RP0 group and the R0 and RP5 groups could not use 17 and 15 chemical compounds (OD < 50 at the end-point), respectively, as single carbon sources. Among chemical compounds, pyruvic acid methyl ester (OD > 100 at the end-point) was used by the bacterial communities from all meat samples, while *L*-asparagine, Tween 40 and *L*-serine were used by those from five meat samples, except CP0, C0 and RP0, respectively. On the other hand, 2-hydroxybenzoic acid could not be used by bacterial communities from all meat samples. α-Cyclodextrin and γ-hydroxybutyric acid could not be used by bacterial communities from all meat samples except the C5 group. 2-Hydroxybenzoic acid, also known as salicylic acid, has an anti-microbial activity [30, 31]. This organic acid is a natural product in the plant tissues [32, 33] and is a common metabolite in plant [34]. 2-Hydroxybenzoic acid can also be synthesized by lactic acid bacteria in fermented food [31, 35]. This acid is therefore often used as a preservative in food and cosmetics to extend the product shelf life [30, 31, 36].

**Fig. 3:**
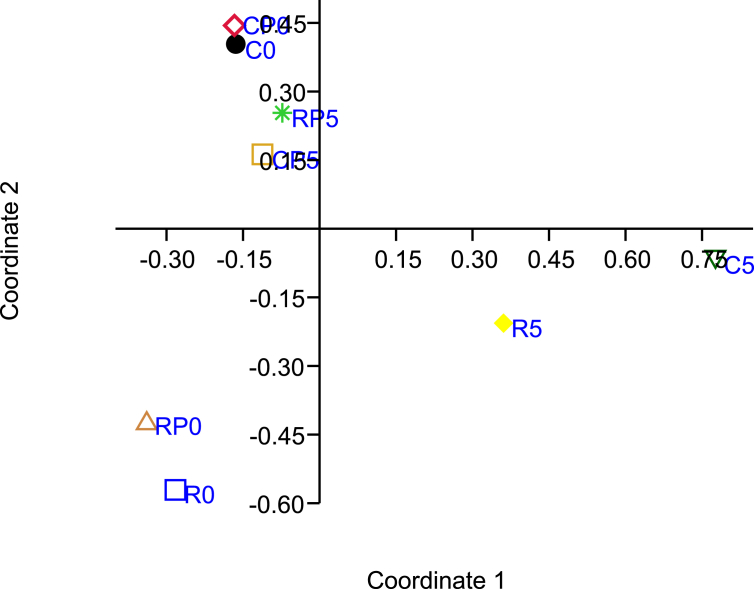
Principal coordinates analysis plot of profile of utilization chemical compounds. The labels of the sample treatments are the same as Fig. 1.

To further determine the functional diversity in bacterial community from poultry ground meat samples, the data from the end-point incubation of meat samples were subjected to principal coordinates analysis (PCoA) with the PAST software [28]. As depicted in Fig. 3, the PCoA plot of the profiles shows the clear separation of bacterial communities from meat samples treated with different methods into three major clusters based on utilization of 31 carbon compounds: C5 and R5, R0 and RP0, and C0, CP0, CP5 and RP5. These results indicate differences of bacterial communities in meat samples after various treatments.

EcoPlates™ have been widely used for analyzing and characterizing the bacterial communities based on carbon utilization patterns from soil and aquatic samples [22, 27, 29]. One critical issue for this methodology is its sensitivity in profiling the bacterial community. A recent study, Sutcliffe et al. [37] have demonstrated that community profiling with physiological assays has a similar sensitivity to the DNA- and RNA-based community profiling. Future studies side by side on both molecular and physiological profiling are needed to ensure the correlation between structure and function in the bacterial communities from meat samples.

In conclusion, the functional profiling of bacterial communities in poultry ground meat samples was assessed by single carbon utilization patterns with EcoPlates™. The results show that the three parameters of the Gompertz growth curves were observed in all samples, 2-hydroxybenzoic acid was not able to be used for a carbon source from all samples, while pyruvic acid methyl ester was used by the bacterial communities from all meat samples, each bacterial community metabolized different numbers of carbon compounds at different rates, and reduction of bacterial functional diversity was observed in the poultry ground meat samples treated with cold plasma and rosemary. In the future, more investigations on whether the physiological profiling in bacterial communities be used as an indicator for effectiveness of cold plasma treatment of meat samples. It is also interesting to explore whether the chemicals not metabolized by the bacterial communities be used as a food preservative.

## Declarations

### Author contribution statement

Hung-Yueh Yeh: Conceived and designed the experiments; Performed the experiments; Analyzed and interpreted the data; Wrote the paper.

John E. Line, Arthur Hinton, Jr.: Performed the experiments; Analyzed and interpreted the data.

Yue Gao, Hong Zhuang: Performed the experiments; Contributed reagents, materials, analysis tools or data.

### Funding statement

This work was supported by the USDA Agricultural Research Service CRIS Project No. 6040-32000-071-00D.

### Competing interest statement

The authors declare no conflict of interest.

### Additional information

No additional information is available for this paper.
